# Network Embedding the Protein–Protein Interaction Network for Human Essential Genes Identification

**DOI:** 10.3390/genes11020153

**Published:** 2020-01-31

**Authors:** Wei Dai, Qi Chang, Wei Peng, Jiancheng Zhong, Yongjiang Li

**Affiliations:** 1 Faculty of Information Engineering and Automation, Kunming University of Science and Technology, Kunming 650050, China; 2 Computer Technology Application Key Lab of Yunnan Province, Kunming University of Science and Technology, Kunming 650050, China; 3 College of Engineering and Design, Hunan Normal University, Changsha 410081, China

**Keywords:** human essential genes, protein–protein interaction network, network embedding, feature representation

## Abstract

Essential genes are a group of genes that are indispensable for cell survival and cell fertility. Studying human essential genes helps scientists reveal the underlying biological mechanisms of a human cell but also guides disease treatment. Recently, the publication of human essential gene data makes it possible for researchers to train a machine-learning classifier by using some features of the known human essential genes and to use the classifier to predict new human essential genes. Previous studies have found that the essentiality of genes closely relates to their properties in the protein–protein interaction (PPI) network. In this work, we propose a novel supervised method to predict human essential genes by network embedding the PPI network. Our approach implements a bias random walk on the network to get the node network context. Then, the node pairs are input into an artificial neural network to learn their representation vectors that maximally preserves network structure and the properties of the nodes in the network. Finally, the features are put into an SVM classifier to predict human essential genes. The prediction results on two human PPI networks show that our method achieves better performance than those that refer to either genes’ sequence information or genes’ centrality properties in the network as input features. Moreover, it also outperforms the methods that represent the PPI network by other previous approaches.

## 1. Introduction

Essential genes are a group of genes that are indispensable for cell survival and cell fertility [1]. Scientists focus on studying essential genes in bacteria to find potential drug targets for new antibiotics [2]. Identifying essential genes in prokaryotic or simple eukaryotic organisms helps us understand the primary requirement for cell life, but also points the way to synthetic biology research that aims to create a cell with a minimal genome. The development of wet-lab experimental techniques, such as single-gene deletions [3], RNA interference [4], and conditional knockouts [5], has accumulated several essential genes in some simple eukaryotic organisms such as *S. cerevisiae* [6], *C. elegans* [7], and *A. thaliana* [8], in the past few years. However, it is hard to apply these techniques to explore human essential genes due to technical difficulties and species limitations [9].

The close relationships between essential genes and human diseases found by previous researches has motivated scientists to identify essential genes in humans to seek guidance for disease treatment [10,11,12]. However, it is hard to apply the computational methods that have successfully predicted essential genes of some simple eukaryotic organisms to humans. The biggest challenge is the lack of known human essential genes, because a human is a high and complex organism, which requires more than basic cell survival and fertility to be completely functional and adapted to the environment. The techniques mentioned above are difficult to use to explore human essential genes correctly. Recently, with the emergence of the CRISPR-Cas gene-editing system, several landmark studies [13,14,15] have individually disrupted every human gene in several cell lines in culture and examined how the loss of each gene affects cell viability by using the gene-editing system. Together, these studies provide the first accurate picture of the sets of genes that are absolutely required for the viability of individual human cell types, a long-awaited analysis that presages efficient screens for tumor-specific essential genes. They have published about 2000 human essential genes [13,14,15] that are indispensable for the viability of individual human cancer cell lines. 

The publication of human essential gene data has motivated researchers to deeply study the functions of human genes. The popular way is to train a machine-learning classifier by inputting the features of some known human essential genes and use the classifier to predict new human essential genes. The outputs are lists of genes with the probabilities to be essential. Then, we set a threshold, i.e., 0.5. If the probabilities are above the threshold, the genes are predicted as essential genes. Guo et al. [16] extracted features from genes’ DNA sequence by a Z-curve method and input these features into an SVM classifier to predict human essential genes. As far as we know, in addition to DNA sequences, there are other biological properties related to the essentiality of genes. Guo’s work inspired us to design a more effective method and extract powerful features to improve the prediction accuracy of human essential genes.

One of the most important features related to the genes’ essentiality is their topology properties in biological networks. The studies on some simple eukaryotic organisms have found that the proteins (genes) with a larger number of interactive partners in the protein–protein interaction (PPI) network are highly likely to be essential proteins (genes), because removing them from the networks would cause the collapse of the network [17]. Therefore, some centrality methods are frequently employed to measure the importance of the genes in the network [9], including degree centrality (DC) [18], closeness centrality (CC) [19], betweenness centrality (BC) [20], information centrality (IC) [21], eigenvector centrality (EC) [22], subgraph centrality (SC) [23] and edge clustering coefficient centrality (NC) [24]. Since the PPI data currently available are incomplete and error prone, some researchers design the composited features by integrating PPI networks with other biological information, such as gene functional annotation data [25], gene expression data (PeC [26], WDC [27], FDP [28]), subcellular information [29], protein domain information [29], and orthologous information [30].

The features mentioned above display the genes’ essentiality from different perspectives. Previous researchers have made great efforts to improve the essential gene prediction accuracy in some simple eukaryotic organisms by employing machine-learning methods and combining some topological and biological features [9]. For example, Chen et al. [6] trained an SVM classifier by the features of some known yeast essential proteins, including the combination of phyletic retention, proteins’ evolutionary rate, paralogs, protein size, and the degree centrality in PPI networks and gene-expression networks. Then, the classifier was adopted to predict other potential yeast essential genes. Zhong et al. [31] developed an XGBoost-based classifier to integrate multiple topology properties of essential genes in the PPI network to predict yeast and *E. coli* essential genes.

As for human essential gene identification, we can draw a lesson from previous studies on other species. However, a machine-learning method that combines multiple biological and topological properties to make predictions still cannot take full advantage of the characteristics of network topology. Genes or their protein products interact with each other to perform their functions in cells together [12,32]. Their interactions in the PPI network play an important role in essential gene prediction. Recently, with the development of biological techniques, the human PPI network becomes even more complete and correct. Therefore, it is imperative to find an effective method to extract the characteristics of genes in the network and represent the human PPI network as much as possible. 

Network embedding is an efficient method to mine the useful information from the network, which converts the network nodes into a vector of low dimensional space while maximally preserves the network structural information and the network properties. There are many network embedding methods [33,34,35,36,37]. In this work, we learn the vector representation of the nodes in the human PPI network by using a random walk-based network embedding method. This kind of method, first, implements random walk on the network to get the network context of a starting node. Then, a word2vec [33] method that originates for natural language processing inputs the node pairs into an artificial neural network and learns the representation vectors for the nodes in the PPI network. The random walk-based network embedding methods different in the way of walking on the network. The DeepWalk [34] method implements a depth-first search on the network, whereas The LINE [35] method implements a breadth-first search strategy to generate the node context. The vertices that share similar neighbors in the network tend to be identical to each other [32]. Hence, in this work, we conduct a bias random walk on the human PPI network to generate the note context, which means a node more likely walks to the connecting nodes sharing similar neighbors with it [38]. After that, the node pairs are input into a neural network to represent the features of genes in the PPI network and, then, these features are put into a variety of classifiers to label whether or not the genes are essential. In contrast to previous supervised machine-learning methods, our method conducts network embedding to extract the features for the genes from the PPI network through mapping them to latent space vectors that maximally preserve the relationships between the nodes and their network neighborhoods. The prediction results on two human PPI networks show that our method achieves better performance that either refers to genes’ sequence information [16] or selects genes’ centrality properties in the network as input features. It also outperforms the methods that represent the PPI network by other previous approaches, i.e., Deepwalk [34] and Line [35]. Part of the contents appeared in the conference paper [39]. In this work, we comprehensively discuss the advantage of bias random walk in the course of network embedding learning, but also investigate what the feature representations of the human essential genes are.

## 2. Methods 

**Figure 1:**
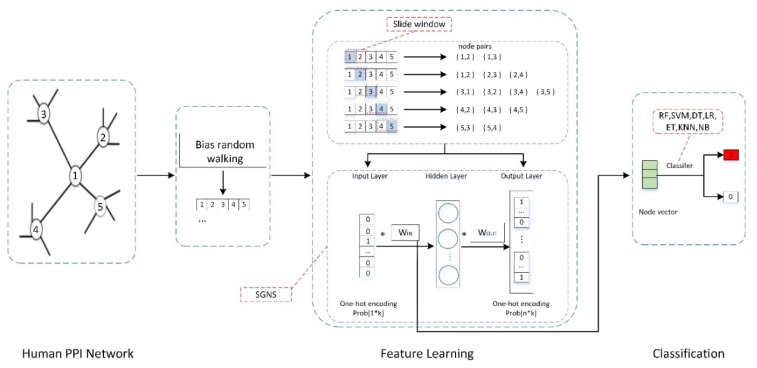
An overview of identifying human essential genes by the network embedding protein–protein interaction (PPI) network.

Our method involved two steps to predict humane essential genes. First, every node in the human PPI network represents a vector that can preserve the structure of the input network. Secondly, the vector is put into a classifier to train the classifier and make predictions. Similar to the node2vec model [38], our method learns the feature representation for every node in the PPI network by the following steps: The first step is to conduct a bias random walk on the PPI network starting from a single node and, then, to obtain a node sequence that records the network context of the starting node. Next, a window slides along the node sequence to get the node pairs that have a close relationship in the network. These node pairs are put into a skip-gram-based architecture to learn the feature representation of every node. Figure 1 shows an overview of our method for identifying essential genes.

### 2.1. Bias Random Walk

Network embedding methods originate for learning features for words in the natural language processing field. The input of the methods is a word sequence that represents the neighborhood relationship of the word in the language context. The word and its neighbor are the input and output layers of an artificial neural network. The weights of the hidden layer are the “node vectors” that we are trying to learn. In this work, the input is a PPI network, which is not linear and is composed of nodes with complicated neighborhood relationships. Hence, to resolve this issue, a bias random walk is simulated on the network to get an ordered node sequence starting from every node in the network. 

Let G = (V,E,W) denote a PPI network, where a vertex v∈ V is a gene and an edge e (u,v) ∈ E connects the genes v and u and w (u,v) ∈ W is the weight of edge e (u,v), which measures the reliability of the connection between u and v. Here, all edges in the PPI network are treated equally, and their weights are set to one. Considering the module nature of the PPI network, a vertex walks to its neighbors at different probabilities. That is, given a vertex v, there is an edge e (u,v), where vertex u is visited by vertex v at the previous step. The vertex v walks to one of its neighbor k by evaluating the transition probabilities T (v,k) on edge e (v,k). The transition probabilities T (v,k) is defined as follows:

T (v,k) = π (u,k) * w (v,x),(1)

$$\mathrm{with}\;\pi \left(u,k\right)\{\begin{array}{c} \frac{1}{p},\;{\;d}_{\mathrm{uk}}=0\\ \;1,{\;d}_{\mathrm{uk}}=1\;\\ \frac{1}{q},{\;d}_{\mathrm{uk}}=2 \end{array},$$

where d_uk_ denotes the shortest path distance from the previous vertex u to the next vertex k. Especially, d_uk_ = 0 means that the vertices u and k are the same vertex and the vertex v jumps back to its previous vertex u, d_uk_ = 1 means that the vertex k is the common neighbor of the vertices u and v, and d_uk_ = 2 means that the u and k connect indirectly, and the vertex k is not their common neighbor. The parameter p controls the likelihood of the vertex v traveling back to its previous vertex u. The parameter q controls the probability of the vertex v jumping to the next vertex k. Setting different values for the parameters p and q guides a bias walk on the network. If the parameters p and q are larger than one, the vertex v is more likely to walk to the vertex that is the common neighbor of it and its previous step, i.e., the vertex u. After computing the transition probabilities for every edge in the PPI network, we row normalize the transition probability matrix T to ensure the sum of the walk-out probabilities for each node to be one.

(2) $${\;T}_{norm}\left(v,k\right)=\frac{T\left(v,\;k\right)}{\sum_{j\in N\left(v\right)}T\left(v,\;j\right)},$$

In bias random walk, a node tends to walk to its neighbor along the edge with the highest transition probability. Suppose a node has number N of neighbors, it usually needs O(N) time to find the walk-out edge with the highest transition probability. This work adopts an alias sampling method to find the edge in O(1) time [38]. The outcome of the bias random walk is a vertex sequence that records the walking trace in the network starting from a vertex, which reserves the neighborhood relationship of the starting vertex in the network. Algorithm 1 gives the pseudocode of the bias random walk.


**Algorithm 1.** Bias random walk algorithm.**Input** G = (V, E, W), Len_walkLists, parameters w, p and q; **Output** vertex sequence lists: walkLists T = computing transition probabilities (G, p, q, w)//computing transition probabilities for every edge in the network T_norm_ = normalizing T by Equation (2) G’ = (V, E, T_norm_) walkLists = {} for iter = 1 to Len_walkLists do    for every node u ∈ V do      Append u to seq      while len(seq) < w:        t = seq [-1] // getting the last node of the set seq        N(t) = sort (GetNeighbors(t, G’)) // sorting neighbor list of current vertex in alphabetic                       order        n = AliasSampling(N(t), T_norm_) //applying alias sampling with respect to the normalized             transition probabilities to select a next visiting neighbor node      Append n to seq    Append seq to walkLists return walkLists


### 2.2. Feature Learning 

After getting the neighbor sequence of every starting vertex in the network by a bias random walk, a window (a parameter c controls the window size) slides along the sequence to get the neighbors of an input vertex. We set a window size two, for example. If the sequence of vertices is {1,2,3,4,5…} and Vertex 1 is the input vertex. The training samples should be {1,2}, {1,3}, {2,1}, {2,3}, {2,4}, {3,2}, {3,1}, {3,4}, {3,5}, {4,3}, {4,2}, {4,5}, {5,4}, and {5,3} (see Figure 1). Then these vertices and their neighbors are input into a skip-gram with negative sampling architecture (SGNS, also called word2vec method) [33] to finish the feature learning for every vertex in the network. Following is a brief overview of the SGNS method.

Give an undirected graph G = (V,E,W), for every vertex v∈V, we can get its sequence of neighbor {*v^m^*} in the network by a bias random walk. Suppose there are number *m* of vertices in the sequence. Let f: v∈V->R^d^ be the function that maps a vertex *v* to its feature representation vector and f(v) denotes the feature representation of the vertex v. Here, d is the size of the feature representation vector, which is empirically set to 64 [33]. Given a vertex v in the input vertex sequence, N(v) denotes its neighborhood set. The SGNS architecture aims to build a model that maximizes the log-probability of neighbors N(v) around a vertex v under the assumption that its feature representation is f (v). The objective function is formally defined as follows:

(3) $$\underset{f}{\max}\sum_{v\in V}\log P(N\left(v\right)|f\left(v\right)),$$

Given the feature representation of the node vertex v, we factorize the likelihood by assuming that the probability of observing a neighbor is independent of observing any other neighborhood node [38].

(4) $$P\left(N\left(v\right)|f\left(v\right)\right)=\underset{u\in N\left(v\right)}{\prod }P(u|f\left(v\right)),\;$$

where P(*u*|f(*v*)) is defined as follows:

(5) $$P\left(u|f\left(v\right)\right)=\frac{\exp\left(f\left(u\right)\cdot f\left(v\right)\right)}{\sum_{v_{s}\in V}\exp\left(f\left(v_{s}\right)\cdot f\left(v\right)\right)},$$

The underlying assumption of this objective function is the vertices that are close in the original network should have similar feature representations in latent space. The SGNS uses a two-layer artificial neural network to train the feature representation of every vertex. Figure 1 shows the workflow of SGNS architecture. Suppose there are m distinct vertices in an input sequence, the SGNS applies an m-dimensional one-hot vector to represent the input sequence. The one-hot vector of the *i*th vertex in an input sequence is a binary vector, whose elements are all zeros except for the *i*th element is one. The input vertex and its neighbors are encoded into corresponding one-hot vectors. Then, these vectors are placed at the input layer and the output layer of the artificial neural network, respectively. The stochastic gradient descent (SGD) technique is adopted to train the weights of the hidden layer. Finally, merely multiplying the 1 × m one-hot vector from left with an m × d weight matrix results in the feature representation vector of an input vertex. Since computing $\underset{v_{s}\in V}{\sum }\exp\left(f\left(v_{s}\right)\cdot f\left(v\right)\right)$ in Equation (4) is too time-consuming for the large scale network, negative sampling is adopted to address the computational problem [33]. 

### 2.3. Classification

The previous steps help us learn feature vectors for genes while maintaining their topological properties in the PPI network. The features of some labeled genes are put into a classifier to build a model. Then, the classifier determines the unlabeled genes to be essential according to their feature vectors. A variety of classifiers are available to finish the prediction task. This work aims to verify that using a network embedding method to learn genes’ features in the PPI network is helpful to predict human essential genes. Hence, one of popular classifiers is selected to predict the human essential genes, including random forest (RF) [40], support vector machine (SVM, RBF kernel) [6], decision tree (DT) [41], logistic regression (LR) [42], extra tree (ET), k-nearest neighbor (KNN) [43] and Naive Bayes (NB) [44].

## 3. Results

### 3.1. Datasets

Two kinds of datasets were involved in this work. The one dataset was the human essential gene dataset, which was from the supplementary files of [16]. The human essential genes came from three recent works [13,14,15], which consist of 1516 essential genes and 10,499 non-essential genes. 

The other dataset was the human PPI network. We tested our method on two different human PPI networks. One network (namely FIs) was downloaded from the Reactome database [45], involving 12,277 genes and 230,243 interactions. The other network was from the InBio Map database (called InWeb_IM dataset) [46], consisting of 17,428 genes and 625,641 interactions. To compare with the Guo’s method [16], the genes which appeared in both the PPI network and the supplementary files of [16] remained in the training and testing datasets. Consequently, the FIs dataset included 1359 essential genes and 5388 non-essential genes, and the InWeb_IM dataset included 1512 essential genes and 9036 non-essential genes. Table 1 shows the details of the two human PPI networks.

### 3.2. Evaluation Metrics 

The five-fold cross-validation was selected to test the prediction performance of our method. All genes in the benchmark dataset partitioned into five parts at random. Four of the five parts were the training set, and the remaining part was the testing set. According to the benchmark dataset, some popular statistical metrics measured the prediction performance. The metrics included precision, recall, Sp (specificity), NPV (negative predictive value), F-measure, ACC (accuracy), and MCC (Matthews correlation coefficient). Their definitions are as follows:

(6) $$Precision=\frac{TP}{TP+FP}\;,$$

(7) $$Recall=\frac{TP}{TP+FN}\;,$$

(8) $$F-measure=\frac{2\cdot precision\cdot recall}{precision+recall}\;,$$

(9) $$Sp=\frac{TN}{TN+FP}\;,$$

(10) $$NPV=\frac{TN}{TN+FN}\;,\;$$

(11) $$MCC=\frac{TP\times TN-FP\times FN}{\sqrt{\left(TP+FP\right)\left(TP+FN\right)\left(TN+FP\right)\left(TN+FN\right)}}\;,$$

(12) $$\mathrm{ACC}=\frac{\mathrm{TP}+\mathrm{TN}}{\mathrm{TP}+\mathrm{TN}+\mathrm{FP}+\mathrm{FN}}\;,\;$$

where TP (true positive) and FP (false positive) measure the number of predicted essential genes that match or not match with known essential genes. FN denotes the number of known essential genes that are missed by predictions. TN indicates the number of true-negative genes. In addition, the area under the Receiver Operating Characteristic (ROC) Curve (AUC) and the area under precision-recall curve (AP) measure the overall performance of each method with selecting different thresholds to get candidate essential genes. 

Due to the unbalance between essential genes and non-essential genes, we took two different strategies in the course of validation. In one strategy, each fold data maintains the same ratio of essential genes to non-essential genes in the original data, i.e., about 1:4 in the FIs dataset and 1:6 in the InWeb_IM dataset. The other strategy keeps the ratio of essential genes to non-essential genes as 1:1 in each fold data. 

### 3.3. Parameter Selection

**Figure 2:**
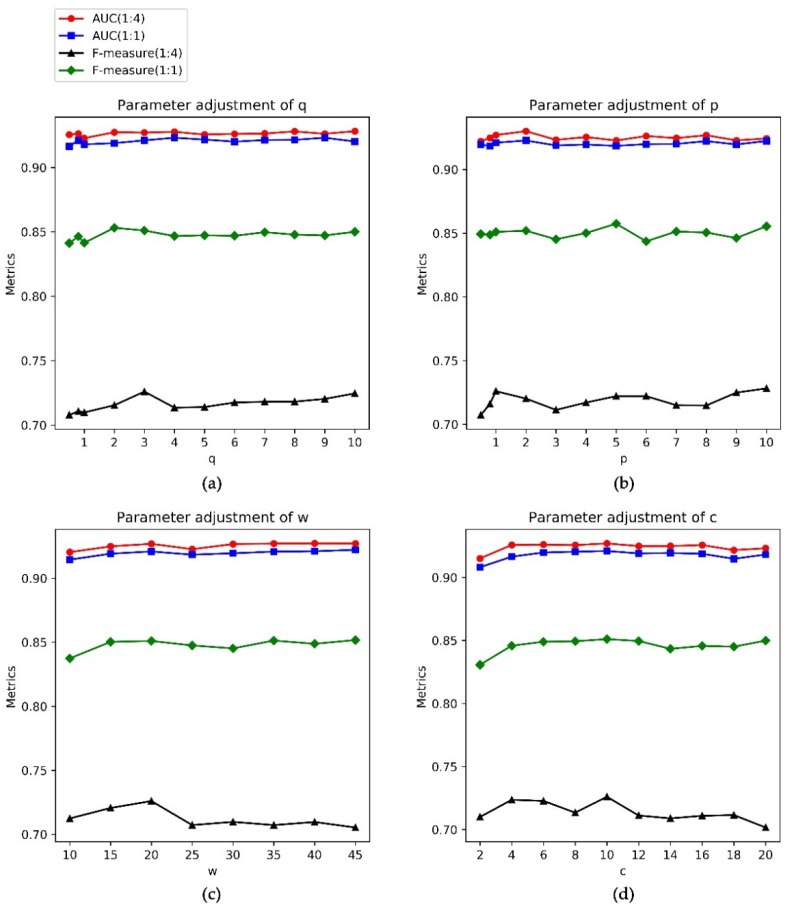
Parameter adjustments on FIs dataset. Different colors indicate different data ratios. (**a**) Fixing the p, w, and c and adjusting q; (**b**) fixing the q, w, and c and adjusting p; (**c**) fixing the p, q, and c and adjusting w; and (**d**) fixing the p, q, and wand adjusting c.

As mentioned in the Method section, the transition probability T (v,k) mainly depends on the value of π (u,k) (see Equation (1)), since, in this work, we only focus on the unweighted human PPI network. Equation (1) shows that the value of π (u,k) changes with different values of the parameters p and q. If the p and q equal one, the node v has an equal probability of jumping back to its previous node u or walking to the common neighbor of it and its last step node u or guiding to its new neighbors. On the one hand, if the p and q exceed one, the node v more likely walks to the node that is the common neighbor of it and its last step node u. On the other hand, if the value of q is less than one, the node has more likelihood of jumping to the nodes far away from it and if the value of p is less than one, the node is more likely to be guided back to its previous nodes. To investigate the effect of parameters on the prediction performance of our method, for the FIs dataset, we fix the values of w, c, and p to 20, 10, and one respectively and set the values of q to 0.5, 0.8 and the integers ranging from one to 10. Figure 2a shows that, when the p and q equal one, the AUC values and F-measure values by keeping the ratio of essential genes to non-essential genes as 1:1 or their original rate on this dataset are 0.9179, 0.9227, 0.8415, 0.7098, respectively. Under this condition, the performance of our method is lower than when the q is 0.8 or ranges from two to six. It suggests that the bias random walk on the human PPI network can improve the performance of human essential gene predictions. Moreover, the AUC values and F-measure values when q ranging from two to 10 vary within a very narrow band and are higher than that when q is less than one. In this work, the q is three on this dataset because the performance of our method receives the best. Similarly, for the parameter p, we fix the values of w, c, and q to 20, 10 and three, respectively, and set the p to 0.5, 0.8 and the integers ranging from one to 10. We notice from Figure 2b, that the AUC values of our method drop quickly when the p is less than one, which suggests that setting more probability to jump back to the previous node reduces the prediction performance. When the parameter p has the same value three as the parameter q, the AUC values are lower than that when the p is one. Under most conditions, setting the p to one achieves better performance than setting p to other values. Hence, in this work, the parameter p is set to one on this dataset.

The parameter w decides the walk distance in the network. The larger the w value is, the farther the node walks away from the start node and the more global information of the network is explored. To investigate the effect of the parameter w, the values of c, p, and q are fixed to 10, one and three, respectively, and the w ranges from 10 to 45 on the FIs dataset. Figure 2c shows that the AUC and the F-measure values of our method achieve relatively high values when the w is 20 on this dataset. Consequently, in the following tests, w was set to 20 on this dataset.

The parameter c controls the size of the sliding window along the node sequence. It decides which two nodes in the node sequence are regarded to have a close relationship in the PPI network and will be the input and output of the artificial neural network. Setting the c values too small ignores many truly related gene pairs and setting the c values too large introduces many unrelated gene pairs. To investigate the effect of the parameter c, we fix the values of w, p, and q to 20, one and three, respectively, and the range of c from two to 20 on the FIs dataset. Figure 2d shows that the AUC values and F-measure values of our method achieve relatively high points when the c is ten on this dataset. Consequently, the c is set to 10 on the FIs dataset in this work.

**Figure 3:**
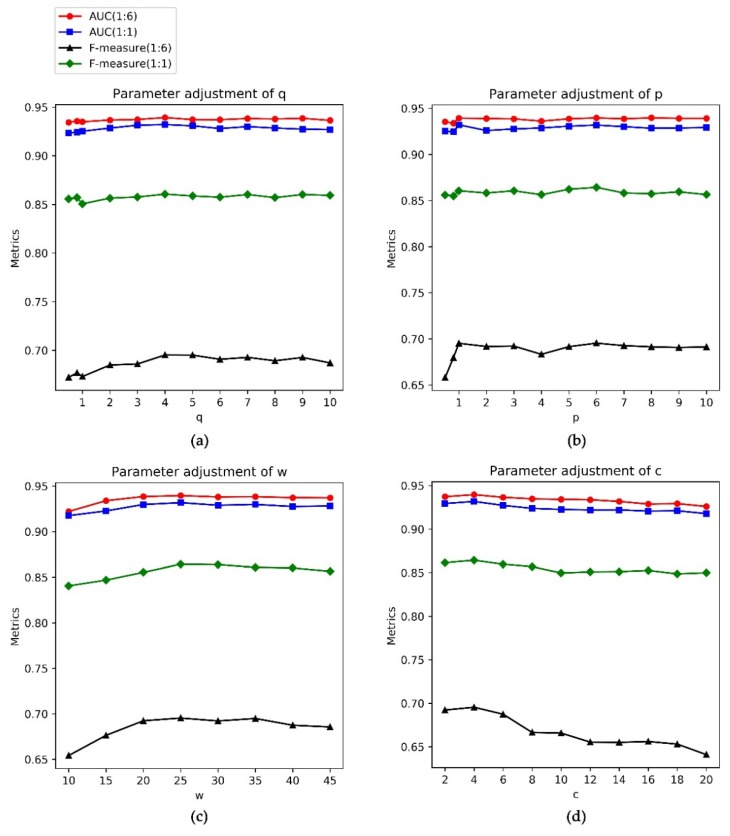
Parameter adjustments on InWeb_IM dataset. Different colors indicate different data ratios. (**a**) Fixing the p, w ,and c and adjusting q; (**b**) fixing the q, w, and c and adjusting p; (**c**) fixing the p, q, and c and adjusting w; and (**d**) fixing the p, q, and w and adjusting c.

For the InWeb_IM dataset, we fixed the values of w, c, and p to 25, four, and one, respectively, and set the q to 0.5, 0.8 and the integers ranging from one to 10. Figure 3a shows that when the values of q equal four, the AUC values and the F-measure values achieve the highest. Hence, the q is four on this dataset. For parameter p, we fixed the values of w, c, and q to 25, four, and four, respectively and set the p to 0.5, 0.8 and the integers ranging from one to 10. Figure 3b shows that setting p to six helps our method achieve the best performance. Similarly, we test the parameters w and c and set the w and c to 25 and four, respectively, on the InWeb_IM dataset. 

### 3.4. Comparison with Existing Methods

To test the effectiveness of our method, we compared it with four other existing approaches, including the Z-curve method [16], the centrality-based method, the DeepWalk [34] method, and the LINE [35] method. The Z-curve method is the latest method to predict human essential genes, which inputs DNA sequence features extracted by a λ-interval Z-curve method into an SVM classifier to make predictions [16]. The centrality-based method makes use of genes’ central indices in the network as input features, including DC, BC, CC, SC, EC, IC, and NC. CytoNCA [47] calculated these centrality indices. The remaining two are the DeepWalk [34] and the LINE [35] that learn the feature representation for every node in the human PPI network by different network embedding methods. All these methods select the SVM classifier to make predictions. Table 2; Table 3 show their prediction performances on two different human network datasets, including the FIs dataset and the InWeb_IM dataset. The ratio of essential genes to non-essential genes in the course of validation shows in the brackets at the first column of the tables. For example, we maintained the ratio of the two kinds of genes as 1:4 on the FIs dataset or 1:6 on the InWeb_IM dataset. The best and comparable results are in bold font.

Table 2 and Table 3 show that our method has the best performance among all the methods compared on the two datasets, no matter maintaining which ratio of essential genes to non-essential genes in the course of validation. When the ratio of the two types of genes was 1:4 on the FIs dataset, the F-measure, MCC, AUC, and AP values of our method were 0.692, 0.641, 0.913, and 0.769, which achieved 31%, 57%, 9.5%, and 47.3% improvements as compared with the Z-curve method and achieved 23%, 38%, 21.2%, and 39.8% improvements as compared with the centrality method. For the comparison with the DeepWalk and LINE, the four indicators showed improvements of 0.6%, 2.6%, 0.9%, 4.8% and 28.4%, 25%, 6.7%, 11%, respectively. When the ratio of the two kinds of genes was 1:6 on the InWeb_IM dataset, the F-measure, MCC, AUC, and AP values of our method were 0.665, 0.641, 0.915 0.762, respectively, which were 40.6%, 65.2%, 8.98%, 67.1% higher than that of the Z -curve method, and were 22.2%, 37.8%, 10.5%, 49.1% higher than that of the centrality method. For the comparison with DeepWalk and LINE, the F-measure, MCC, AUC, AP values of our method showed improvements of 9%, 12.3%, 1.2%, 14.2%, and 141.8%, 93.1%, 9.8%, 39.3%, respectively. When the ratio of the two kinds of genes was kept as 1:1 in the course of cross-validation, the evaluation metrics of most comparing methods, including precision, recall, F-measure, ACC, MCC, AUC, and AP, increase their values to some degree. Compared with DeepWalk that has the highest performance among the other existing methods, the F-measure, MCC, AUC and AP values of our method were 0.847, 0.699, 0.914, 0.902 on the FIs dataset and reached up to 0.857, 0.713, 0.928, 0.921 on the InWeb_IM dataset. Our method still possesses the highest F-measure, MCC, AUC, and AP values on the two datasets.

The Z-curve method used the gene’s sequence information as prediction features. The centrality method combined seven genes’ central indices in the network into feature vectors to make predictions. The success of our method proved that learning the genes’ latent features from the PPI network, which extremely maintains the structure of the network, was able to improve the identification of human essential genes. Compared with DeepWalk and LINE that also extracted genes’ features by network-embedding, our methods showed better performance, which proves that our method takes better network-embedding strategies to predict human essential genes. 

### 3.5. Comparison of Different Classifiers

In this work, we extract the gene’s features in the PPI network by using a network-embedding method. To assess the effectiveness of the features on human essential gene predictions, besides the SVM classifier, the other six frequently used classifiers are selected to predict human essential genes in our method. These classifiers include deep neural network (DNN), decision tree (DT), Naive Bayes (NB), k-nearest neighbor (KNN), logistic regression (LR), random forest (RF), and extra tree (ET). The batch_size, epochs, learning rate, and activation function of the DNN model are 64, 20, 0.0001, and sigmoid, respectively. For the DNN with one hidden layer, the number of neurons is 32. For the DNN with three hidden lays, the numbers of neurons are 32, 16, and one, respectively. Table 4 and Table 5 list the performance comparisons of our method on two different datasets with different classifiers and different ratios of essential genes to non-essential genes in cross-validation. Among all comparing classifiers, the extra tree algorithm (ET) based on random achieved the highest prediction performance. By maintaining the original essential and non-essential gene ratio in the training and testing dataset, the F-measure, MCC, AUC, and AP values of our method by using ET classifier achieved 0.727, 0.676, 0.932, 0.806 on the FIs dataset, and 0.692, 0.659, 0.943, 0.779 on the InWeb_IM dataset. When keeping the same ratio of essential gene to non-essential, the F-measure, MCC, AUC, and AP values of our method using ET classifier are 0.7%, 1.9%, 0.98%, and 2.2% higher than that by using the SVM classifier on the FIs dataset, and are 1.05%, 2.2%, 0.65%, and 0.76% higher than that by using the SVM classifier on the InWeb_IM dataset. The results suggest that choosing other efficient classifiers can further improve the prediction performance of our method. 

### 3.6. Feature Representation of Human Essential Genes

Our method represents every node in the human PPI network as a latent vector and uses the vector as the features of the node to predict essential proteins. To investigate whether the feature representation learned by our method can preserve network structure and the properties of the nodes in the network, we rebuild the network according to the Euclidean distance between the nodes’ feature vectors. Then, we calculate some centrality indexes for nodes in the PPI network, including DC, BC, CC, NC, and IC. Table 6 shows the Pearson's correlation coefficient between these centrality indexes of the human essential genes in the original PPI network and the rebuilt network. We note that on the FIs dataset and InWeb_IM dataset, the Pearson's correlation coefficients between the centrality indexes of the human essential genes in the original and the rebuilt network are above 0.8 and some values even close to one. It suggests the essential genes have very similar topology properties in the two PPI networks and the feature representation learned by our method can preserve network structure. 

**Figure 4:**
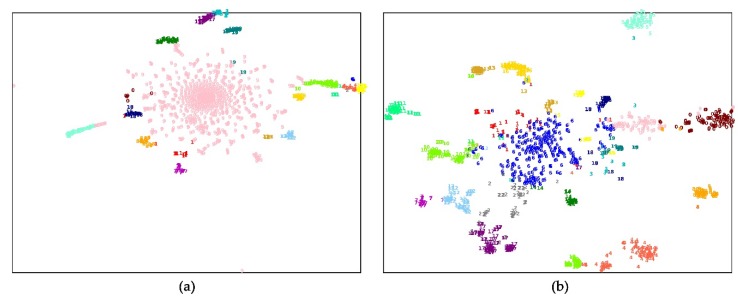
The k-means clustering essential genes by two different features on the FIs dataset. (**a**) By connection relationship features; (**b**) by feature representation.

**Figure 5:**
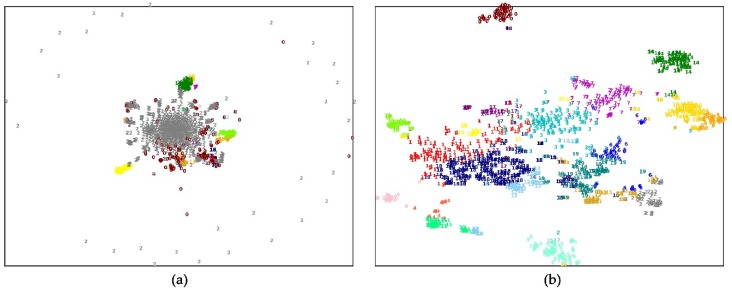
The k-means clustering essential genes by two different features on the InWeb_IM dataset. (**a**) By connection relationship features; (**b**) by feature representation.

To probe why the feature representations can improve the performance of the human essential genes’ prediction, we clustered the essential genes into 20 subgroups according to their features by a k-means method. For comparison, we select two different kinds of features for clustering. One type of gene feature is the connection relationship between a gene and the other genes in the PPI network, which is a row of the adjacent matrix of the network. The other type of gene feature is a gene’s feature representation learned by the network embedding method. The clustering results on two human PPI networks were visualized by a t-SNE tool. Figure 4; Figure 5 show that the human essential genes can be separated into several subgroups well, when using the genes’ feature representations. On the FIs dataset, the largest subgroup consists of 289 essential genes, and the smallest one consists of 21 essential genes. On the InWeb_IM dataset, the largest subgroup consists of 176 essential genes, and the smallest one consists of 20 essential genes. However, when the clustering features are selected as the connection relationship between a gene and the other genes in the PPI network, most of the human essential genes are aggregated in a large subgroup while the remaining subgroups have small sizes. On the FIs dataset, the largest subgroup consists of 902 essential genes, and the smallest one consists of six essential genes. On the InWeb_IM dataset, the largest subgroup consists of 1022 essential genes, and the smallest one consists of one essential gene. Moreover, we also leverage silhouette value and Dunn value to evaluate the quality of the clustering results under two different clustering features. Table 7 lists the detailed information about these subgroups. The silhouette and Dunn values of the subgroups generated by the feature representation are higher than that of the subgroups generated by the connection relationship features. It suggests that selecting the feature representation as clustering features can successfully divide human essential genes into the subgroups with the high compactness within clusters and the high separation among clusters. 

To further investigate the biological functions of these essential gene subgroups, we used the DAVID on-line database to perform the GO functional enrichment analysis for these subgroups in the GOTERM_BP_FAT category. In this work, we only present the enrichment analysis based on the GO biological process (BP) annotation. Because the module enriches in BP annotations indicates that the genes in the module have diverse molecular functions but work together to perform a particular biological process, such as signaling in a pathway. An essential gene subgroup is regarded as being biological significance if its *p*-value is less than 0.01. All of the essential gene clusters generated by two different clustering features on the two human PPI networks have significant biological functions (see Supplementary Files). As we can see from Table 7, the essential gene subgroups clustered by the feature representation have higher vital biological functions (with higher Avg(-log(*p*-value)) values) than those gathered by connection relationship features. Table 8 lists ten example essential gene clusters with the smallest *p*-values on the FIs dataset. We notice that the feature representation can cluster a group of essential genes with the *p*-value of 6.64E-191. It consists of 116 essential genes, and 110 of them enrich in the function of RNA splicing (GO:0000377). A cluster includes 50 essential genes, and all of the genes in it have the function of rRNA processing (GO:0006364). However, these 50 essential genes are separated into three clusters by connection relationship features (see Supplementary Files). The bias random walk in the course of learning feature representation for genes can catch the modularity structure of the network. Therefore, these features contribute to the performance improvement of essential gene prediction because essential genes tend to cluster together.

## 4. Conclusions

Extracting the features related to essential genes is the critical step for designing a machine-learning method with powerful prediction performance. Compared with previous approaches that refer to the topological features in the PPI network or learn features from the DNA sequence, this work adopts a network embedding method to represent the nodes in the PPI network as latent feature vectors that maximally preserve their interactive relationships in the network. To measure the power of the latent features on predicting human essential proteins, we learn features from two different human PPI networks and these features are input into several popular classifiers. In the course of learning, a bias random walk is, first, implemented on the PPI network to get the network context of a given node and, then, the node pairs in the network context are input into an artificial neural network to learn their representation in the latent space. The human essential gene prediction results based on the two PPI networks show that our method using the SVM classifier outperforms the methods [16] that select network topological properties or the DNA sequences as input features. It proved that the network embedding method could learn feature vectors for the nodes from the PPI network to efficiently predict human essential genes. The features represented by our method have better performance on human essential gene prediction than that by other network embedding approaches, i.e., Deepwalk [34] and LINE [35]. It suggests that our approach is an efficient way to learn the feature representations for nodes in the human PPI network by adding bias in exploring neighborhoods. Moreover, using the ET classifier can further improve the prediction performance of our method.

In the future, we will focus on designing a more robust network embedding method to find the latent feature representation of the nodes in the PPI network, and developing more effective machine learning methods to predict human essential genes based on the features of genes. In addition, one human gene can produce many different protein isoforms and there exists noise in the human PPI network. Hence, in future work, establishing reliable gene–gene networks would be a potential solution to improve the essential gene prediction.

## Figures and Tables

**Table 1:** Details of two human PPI networks.

Data set	Genes	Interactions	Essential genes	Training and testing genes
FIs	12277	230243	1359	6747
InWeb_IM	17428	625641	1512	10548

**Table 2:** Performance comparison between our method and existing methods with different essential to non-essential gene ratios on FIs dataset.

Methods	Precision	Recall	SP	NPV	F-measure	MCC	ACC	AUC	AP
DeepWalk (1:4)	0.771	**0.621**	0.954	**0.909**	0.688	0.625	0.887	0.905	0.734
LINE (1:4)	**0.837**	0.394	**0.981**	0.866	0.539	0.513	0.863	0.856	0.693
Centrality (1:4)	0.608	0.523	0.917	0.879	0.562	0.464	0.836	0.753	0.550
Z-curve (1:4)	0.526	0.530	0.880	0.882	0.528	0.409	0.810	0.834	0.522
Our method (1:4)	0.822	0.597	0.968	0.905	**0.692**	**0.641**	**0.893**	**0.913**	**0.769**
DeepWalk (1:1)	0.833	0.846	0.830	**0.843**	0.839	0.676	0.838	0.907	0.896
LINE (1:1)	0.705	**0.847**	0.647	0.808	0.770	0.503	0.747	0.839	0.848
Centrality (1:1)	0.852	0.553	**0.904**	0.669	0.671	0.488	0.728	0.760	0.797
Z-curve (1:1)	0.733	0.800	0.708	0.780	0.765	0.511	0.754	0.824	0.783
Our method (1:1)	**0.859**	0.836	0.863	0.840	**0.847**	**0.699**	**0.849**	**0.914**	**0.902**

**Table 3:** Performance comparison between our method and existing methods with different essential to non-essential gene ratios on InWeb_IM dataset.

Methods	Precision	Recall	SP	NPV	F-measure	MCC	ACC	AUC	AP
DeepWalk (1:6)	0.749	0.515	0.971	0.923	0.610	0.571	0.906	0.904	0.667
LINE (1:6)	0.819	0.165	**0.994**	0.877	0.275	0.332	0.875	0.832	0.547
Centrality (1:6)	0.527	**0.562**	0.916	0.926	0.544	0.465	0.865	0.828	0.511
Z-curve (1:6)	0.485	0.462	0.918	0.911	0.473	0.388	0.853	0.840	0.456
Our method (1:6)	**0.841**	0.550	0.983	**0.929**	**0.665**	**0.641**	**0.921**	**0.915**	**0.762**
DeepWalk (1:1)	0.817	0.848	0.811	0.842	0.832	0.659	0.829	0.908	0.894
LINE (1:1)	0.641	**0.905**	0.492	0.839	0.750	0.436	0.699	0.804	0.792
Centrality (1:1)	0.816	0.713	0.839	0.745	0.761	0.557	0.776	0.851	0.841
Z-curve (1:1)	0.730	0.777	0.713	0.762	0.753	0.491	0.745	0.827	0.801
Our method (1:1)	**0.855**	0.858	**0.854**	**0.858**	**0.857**	**0.713**	**0.856**	**0.928**	**0.921**

**Table 4:** Performance comparison of our method with different classifiers and different essential and non-essential gene rates on FIs dataset.

Methods	Precision	Recall	SP	NPV	F-measure	MCC	Accuracy	AUC	AP
DNN (1 layer, 1:4)	0.258	0.770	0.220	0.805	0.234	0.026	0.667	0.460	0.242
DNN (3 layers, 1:4)	0.248	0.407	0.690	0.822	0.308	0.083	0.633	0.534	0.245
DT (1:4)	0.623	0.619	0.906	0.904	0.621	0.526	0.848	0.763	0.660
NB (1:4)	0.553	**0.737**	0.850	**0.928**	0.632	0.531	0.827	0.880	0.683
KNN (1:4)	0.795	0.628	0.959	0.911	0.702	0.644	0.893	0.889	0.782
LR (1:4)	0.787	0.585	0.960	0.902	0.671	0.613	0.885	0.914	0.755
SVM (1:4)	0.822	0.597	**0.968**	0.905	0.692	0.641	0.893	0.913	0.769
RF (1:4)	0.826	0.646	0.966	0.916	0.726	0.674	0.902	0.927	0.799
ET (1:4)	**0.828**	0.648	0.966	0.916	**0.727**	**0.676**	**0.902**	**0.932**	**0.806**
DNN (1 layer, 1:1)	0.554	0.452	0.503	0.504	0.527	0.007	0.503	0.500	0.519
DNN (3 layers, 1:1)	0.535	0.538	0.532	0.536	0.536	0.070	0.535	0.562	0.553
DT (1:1)	0.768	0.788	0.762	0.783	0.778	0.551	0.775	0.775	0.831
NB (1:1)	0.822	0.765	0.834	0.780	0.792	0.600	0.799	0.876	0.873
KNN (1:1)	0.837	0.805	0.844	0.812	0.821	0.649	0.824	0.895	0.906
LR (1:1)	0.839	0.827	0.841	0.829	0.833	0.668	0.834	0.910	0.907
SVM (1:1)	0.859	0.836	0.863	0.840	0.847	0.699	0.849	0.914	0.902
RF (1:1)	0.859	**0.844**	0.861	**0.846**	0.851	0.705	0.852	0.921	0.921
ET (1:1)	**0.867**	0.840	**0.872**	0.845	**0.853**	**0.712**	**0.856**	**0.923**	**0.922**

**Table 5:** Performance comparison of our method with different classifiers and different essential and non-essential gene rates on InWeb_IM dataset.

Methods	Precision	Recall	SP	NPV	F-measure	MCC	Accuracy	AUC	AP
DNN (1 layer, 1:6)	0.355	0.772	0.206	0.877	0.261	0.103	0.712	0.499	0.206
DNN (3 layers, 1:6)	0.350	0.255	0.921	0.881	0.295	0.202	0.826	0.483	0.221
DT (1:6)	0.584	0.588	0.930	0.931	0.586	0.517	0.881	0.759	0.616
NB (1:6)	0.456	0.697	0.861	**0.945**	0.551	0.473	0.837	0.877	0.615
KNN (1:6)	0.778	0.564	0.973	0.930	0.654	0.617	0.914	0.888	0.740
LR (1:6)	0.785	0.591	0.973	0.934	0.675	0.637	0.918	0.931	0.749
SVM (1:6)	**0.841**	0.550	**0.983**	0.929	0.665	0.641	0.921	0.915	0.762
RF (1:6)	0.799	**0.615**	0.974	0.938	**0.695**	0.659	0.923	0.940	0.776
ET (1:6)	0.816	0.600	0.977	0.936	0.692	**0.659**	**0.925**	**0.943**	**0.779**
DNN (1 layer, 1:1)	0.652	0.504	0.568	0.591	0.607	0.157	0.578	0.603	0.640
DNN (3 layers, 1:1)	0.737	0.497	0.823	0.620	0.593	0.338	0.659	0.637	0.692
DT (1:1)	0.802	0.791	0.805	0.794	0.797	0.596	0.798	0.798	0.849
NB (1:1)	0.836	0.708	0.862	0.747	0.767	0.576	0.785	0.874	0.872
KNN (1:1)	0.853	0.843	0.854	0.845	0.848	0.697	0.849	0.904	0.906
LR (1:1)	**0.865**	0.834	**0.870**	0.840	0.849	0.704	0.852	0.925	0.920
SVM (1:1)	0.855	0.858	0.854	0.858	0.857	0.713	0.856	0.928	0.921
RF (1:1)	0.844	**0.886**	0.836	**0.880**	0.864	0.723	0.861	0.932	0.920
ET (1:1)	0.853	0.879	0.849	0.876	**0.866**	**0.729**	**0.864**	**0.934**	**0.928**

**Table 6:** The Pearson's correlation coefficient between the centrality indexes of the human essential genes in the original PPI network and the rebuilt network.

Data set	DC	BC	CC	NC	IC
FIs	0.9262	0.8040	0.9998	0.9911	0.9794
InWeb_IM	0.9617	0.8372	0.9999	0.9938	0.9839

**Table 7:** Details of the subgroups aggregated by two different features.

Data Set	Features	Max Size	Min Size	Median Size	Silhouette	Dunn	Avg(-log(*p*-value))
FIs	Feature representation	289	21	51.5	0.3242	0.59	63.50
	Connection relationship	902	6	19.5	0.2448	0.58	45.01
InWeb_IM	Feature representation	176	20	61.5	0.2279	0.63	54.95
	Connection relationship	1022	1	13	0.1619	0.25	30.11

**Table 8:** Ten example essential gene clusters with the smallest *p*-values gathered by feature representation on FIs dataset.

GO ID	Description	*p*-value	Genes in Cluster	Gene Ratio
GO:0000377	RNA splicing, via transesterification reactions with bulged adenosine as nucleophile	6.64·10^−191^	*PLRG1/PABPN1/SNRNP35/HNRNPM/RBMX/RBM22/DHX9/MAGOH/XAB2/SRRM2/SNRPF/SMNDC1/SRRM1/SF3B5/PPIE/CTNNBL1/PRPF40A/SNRNP70/PRPF4B/EIF4A3/PRPF19/BUD31/HNRNPL/NCBP1/DHX8/SNRPC/CWC22/CPSF1/RNPC3/HNRNPC/TFIP11/SNU13/CLP1/SNRNP200/ISY1/TXNL4A/CPSF2/USP39/SNRNP27/ALYREF/* *DDX23/CPSF3/PCBP1/DDX39B/PCF11/SYMPK/SF3B1/BCAS2/SRSF1/SF3A2/WDR33/SRSF11/DHX38/SNRNP25/SNRPE/HNRNPH1/ZMAT5/RBM17/SNRNP48/CHERP/PUF60/FIP1L1/HNRNPK/HNRNPA2B1/CSTF3/LSM7/CDC40/SNRPD3/PRPF3/PRPF31/NUDT21/SART3/AQR/CRNKL1/U2AF2/PPWD1/PRPF6/SRSF2/LSM4/SRRT/DHX16/SART1/CSTF1/SF1/SRSF7/SNRPB/DHX15/EFTUD2/NCBP2/PRPF4/SF3B2/SLU7/CDC5L/SF3A3/LSM2/GPKOW/SUGP1/HNRNPU/SF3B4/CPSF4/PRPF8/SRSF3/SNRPD2/HSPA8/SNW1/U2SURP/DDX46/SF3A1/SF3B3/PDCD7*	110/116
GO:0070125	mitochondrial translational elongation	6.97·10^−158^	*MRPL28/MRPL22/MRPL17/MRPL48/MRPL37/MRPL4/MRPL47/TUFM/MRPL23/MRPL9/MRPL24/MRPS12/MRPL11/MRPL10/MRPL38/ERAL1/MRPL46/MRPS6/MRPS25/MRPL41/MRPS27/MRPL12/MRPS16/MRPS23/MRPL34/MRPS34/MRPL43/MRPL15/MRPS24/MRPL35/MRPL40/MRPL57/MRPL21/GFM1/MRPS7/PTCD3/MRPS22/MRPL13/MRPL51/MRPL53/MRPS2/MRPL14/MRPS5/MRPL45/MRPL18/DAP3/AURKAIP1/MRPL19/MRPS15/MRPL20/MRPL39/MRPL44/GADD45GIP1/MRPS18A/MRPS14/MRPS11/MRPS31/MRPS18C/MRPS18B/MRPS30/MRPL16/MRPS35/MRPS10/MRPL33*	64/67
GO:0000184	nuclear-transcribed mRNA catabolic process, nonsense-mediated decay	7.98·10^−107^	*UPF1/RPS16/RPSA/SMG5/RPS14/RPL10A/RPL8/RPS11/SMG7/RPL13A/RPLP1/ETF1/RPL27A/EIF4G1/GSPT1/RPLP2/RPL4/RPS13/RPL18/RPS4X/RPL36/RPS10/RPL23A/RPS12/RPS5/RPL9/SMG6/RPS18/RPS21/RPS7/RPL29/RPL31/RPL12/RPL10/RPL7/PABPC1/RPL3/RPS15A/RPL37A/RPL18A/RPL19/RPS25/SMG1/RPL11/RPL7A/RPS15/RPS9/RPS2/UPF2/RPL27/RPL13/RPL14/RPL15/RPS29/RPL38/RPLP0/RPS8/RPS6*	58/96
GO:0000819	sister chromatid segregation	1.97·10^−94^	*PMF1/NSL1/BUB3/ESPL1/NUP43/BIRC5/SMC2/XPO1/CENPC/NUF2/KIF18A/SEH1L/SKA1/NUP62/SEC13/NCAPD3/NCAPD2/TTK/MAU2/SMC5/NSMCE2/CENPA/CDCA8/RAD21/AHCTF1/CENPM/RANGAP1/SPC24/PLK1/SMC1A/AURKB/CENPK/INCENP/RANBP2/NUP85/SPC25/CKAP5/NUP107/CENPE/SMC3/NUP98/CENPL/PAFAH1B1/MAD2L1/CENPN/SPDL1/NDC80/CDC20/DSN1/BUB1B/NUP133/TPR/KPNB1/CENPI/SMC4/NUDC/SGO1/CCNB1/RAN/NUP160/CDCA5*	61/91
GO:0006364	rRNA processing	5.08·10^−94^	*WDR75/BMS1/EXOSC9/UTP6/NOP56/HEATR1/EXOSC1/UTP20/UTP4/UTP14A/EXOSC8/RRP36/NOL6/MPHOSPH10/DDX47/RRP9/TBL3/PWP2/EXOSC7/WDR3/WDR18/IMP4/EXOSC5/EXOSC4/WDR46/UTP18/DIEXF/UTP11/LAS1L/UTP3/UTP15/NOC4L/FBL/IMP3/NOP14/PDCD11/TEX10/NOP58/RCL1/XRN2/KRR1/DDX49/EXOSC3/EXOSC10/WDR43/DDX52/EXOSC6/NOL11/EXOSC2/WDR36*	50/50
GO:0031145	anaphase-promoting complex-dependent catabolic process	5.71·10^−89^	*ANAPC11/PSMD7/PSMD14/PSMA7/PSMB3/PSMB6/PSMA3/PSMD12/PSMA4/PSMB5/PSMA2/ANAPC2/PSMB1/ANAPC4/PSMD6/ANAPC5/PSMA5/PSMD2/PSMD4/PSMC6/PSMC1/CDC16/ANAPC15/PSMD1/PSMD11/PSMB4/PSMD3/PSMD8/CUL3/ANAPC10/PSMC4/PSMA1/PSMC2/PSMC3/PSMB7/AURKA/PSMC5/PSMD13/PSMB2/CDC23*	40/51
GO:0098781	ncRNA transcription	2.19·10^−81^	*GTF2E1/PHAX/INTS5/TAF13/TAF6/POLR2E/ZC3H8/ELL/POLR2H/TAF8/INTS7/POLR2F/BRF1/GTF2A1/POLR2B/SNAPC2/POLR2I/GTF3C2/INTS9/TAF5/INTS8/GTF2A2/INTS2/POLR2G/POLR2C/GTF3C4/INTS3/SNAPC4/GTF3C3/GTF2B/POLR2D/INTS6/SNAPC3/INTS1/CCNK/CDK9/ICE1/POLR2L/RPAP2/GTF3C5/INTS4/CDK7/GTF2E2/GTF3C1*	44/80
GO:0006270	DNA replication initiation	2.12·10^−66^	*CDC7/MCM2/POLA1/MCM4/POLE2/ORC5/ORC2/CDC45/MCM3/ORC4/MCM7/ORC3/MCM10/CDC6/MCM6/ORC6/ORC1/MCM5/PRIM1/POLA2/GINS2/CDT1/CDK2/GINS4/POLE*	25/28
GO:0048193	Golgi vesicle transport	1.46·10^−61^	*GBF1/TRAPPC1/VPS52/COG4/KIF11/COPB1/DCTN5/VPS54/YKT6/DCTN6/STX18/COPA/COPB2/NSF/COG8/PREB/TMED2/TMED10/TRAPPC4/DCTN4/KIF23/COPG1/TRAPPC5/ARFRP1/ARCN1/NAPA/COPE/TRAPPC8/RINT1/SCFD1/COPZ1/STX5/TRAPPC11/SYS1/NBAS/COG3/SEC16A/TRAPPC3/RACGAP1/GOSR2*	40/ 47
GO:0042254	ribosome biogenesis	5.70·10^−53^	*WDR12/FTSJ3/NOP2/DDX56/MAK16/BRIX1/RRP1/NAT10/ABCE1/GTPBP4/PPAN/MDN1/PES1/EBNA1BP2/DIS3/NOP16/POP4/DDX27/EFL1/SDAD1/NSA2/HEATR3/RPF1/RSL1D1/RRS1/TSR2/TRMT112/LSG1/NHP2/NOP10/NOL9/DKC1/NIP7/GNL2/ISG20L2/RPL7L1/SURF6/DDX51/RRP15/PELP1/NGDN/NOC2L/WDR74*	43/ 81

## Supplementary Materials

The following are available online at https://www.mdpi.com/2073-4425/11/2/153/s1, Table S1: GO enrichment analysis for the essential gene subgroups clustered by the feature representation on the FIs dataset, Table S2: GO enrichment analysis for the essential gene subgroups clustered by the connection relationship features on the FIs dataset, Table S3: GO enrichment analysis for the essential gene subgroups clustered by the feature representation on the InWeb_IM dataset, Table S4: GO enrichment analysis for the essential gene subgroups clustered by the connection relationship features on the InWeb_IM dataset.
